# A rapid and sensitive method to assess seed longevity through accelerated aging in an invasive plant species

**DOI:** 10.1186/s13007-020-00607-3

**Published:** 2020-05-08

**Authors:** Erola Fenollosa, Laia Jené, Sergi Munné-Bosch

**Affiliations:** Department of Evolutionary Biology, Ecology and Environmental Sciences, and Institute of Research in Biodiversity (IRBio), University of Barcelona, Barcelona, Spain

**Keywords:** Aizoaceae, Deterioration, Germination, Management, Post-eradication, Seed survival curve, Soil seed bank, Solarization, Temperature, Thermotolerance

## Abstract

**Background:**

Seed longevity and vigor assessment is crucial for efficient ex situ biodiversity conservation in genebanks but may also have potential applications for the understanding of ecological processes and in situ biodiversity conservation. In fact, one of the factors determining the persistence of invasive species, a main threat to global biodiversity, is the generation of soil seed banks where seeds may remain viable for several years. Artificial seed aging tests using high temperatures and high relative humidity have been described for seed longevity estimation but have been mainly optimized for species with commercial interest. Thus, the aim of the study is to define a rapid and sensitive method to assess seed longevity and vigor through accelerated aging in the worldwide distributed invasive species *Carpobrotus edulis* to provide tools to biodiversity managers to evaluate invasive potential and develop effective post-eradication plans.

**Results:**

Slow seed deterioration rate was obtained when *C. edulis* seeds were subjected to common accelerated aging temperatures (43–45 °C). This contrasts with the rapid viability decay between 24–72 h when seeds were subjected to temperatures superior to 55 °C, a strong inflection point for this species’ thermosensitivity. Relative humidity also played a role in defining seed survival curves, but only at high temperatures, speeding up the deterioration process. The selected aging conditions, 55 °C at 87% relative humidity were tested over two *C. edulis* populations and three measures were proposed to parametrize the differential sigmoidal seed survival curves, defining the seed resistance to deterioration (L_5_, aging time where 95% of seeds maintain their viability), medium longevity (L_50_, 50% of seeds lose their viability) and lethal aging time (L_95_, 95% of viability loss).

**Conclusions:**

An accelerated aging test at 55 °C and 87% relative humidity constitutes a rapid and sensitive method that can be performed within a working week, allowing managers to easily test seed vigor and longevity. This test may contribute to assess invasive potential, design effective monitoring programs and soil seed bank eradication treatments.

## Background

Seed longevity is described as the period over which a seed remains viable and capable of germination [1]. The length of time during which seeds maintain their viability and vigor is a trait of interest not only for crop species within breeding programs and food security, but also for describing natural populations’ dynamics and assess persistence potential [2–4]. Seed longevity is strongly variable, ranging from just a few months in soybean [5], to more than 2000 years in *Phoenix dactylifera* [6]. In species with orthodox seeds, viable seeds may enter dormancy, a temporal suppression of germination under favorable conditions, thanks to their ability to tolerate considerable desiccation [7]. The slow pace of seed viability loss in orthodox species hinders the study and characterization of species’ seed longevity under natural conditions. Due to those difficulties, ex situ aging tests are used to estimate seed longevity and vigor [8]. Genetic factors have a key influence on seed persistence and because of this, the survival of seeds experiencing moisture and temperature stress in the laboratory is thought to be a good predictor of their potential persistence in the environment [1]. In fact, similar molecular events were found to accompany artificial and natural seed aging processes [9]. Long et al. [10] successfully predicted species seed persistence in the field with the species ranking of the days taken for seed viability to decline by 50% in an artificial aging test. A faster viability decay under artificial aging conditions is linked to a germination depression with time [11]. There are different proposed approaches to estimate and contrast seed longevity by generating an atmosphere that speeds up the aging process: accelerated aging and controlled deterioration. Controlled deterioration tests were first developed to identify low vigor seeds [12] and consist of subjecting to high temperatures (around 40–45 °C) prearranged high seed moisture content (from 18 to 24%) for 24 to 96 h [13]. In contrast, accelerated aging (AA) tests consist of exposing seeds to high temperatures around 40–45 °C over water, generating an atmosphere with high relative humidity (RH). The AA test was first developed by Delouche and Baskin [14] to estimate the longevity of seeds stored in a warehouse, as the decline in germination following AA is proportional to the initial physiological potential of the seed lot. Seed lots with high vigor should be able to withstand these stress conditions and will deteriorate at a slower rate than lots with poorer vigor [8]. The AA test is preferable for a rapid method as it does not require initial seed moisture control and equilibration, which may suppose extra time and offers similar results to the controlled deterioration test [13].

Standard AA conditions proposed for several crop seeds varies between 41 and 43 °C for 48 to 72 h [15]. However, even within a species, seed longevity in AA tests may depend on several factors such as moisture content, RH, oxygen partial pressure and temperature [16]. Higher temperatures and high RH are known to boost seed viability loss [11] but may have different effects in different species. Artificially aged seeds of rice lose their viability between 25 to 45 days at 40 °C and 80% of RH but just in 6 days at 45 °C and 100% RH, whereas *Arabidopsis thaliana* seeds lose their viability within 3 days at 37 °C at 83% RH [8]. Artificially aging protocols have been mainly designed for grain, vegetable, forage, and forestry crops [17] such as rice, wheat, soybean and tomato, leaving aside other species with ecological interest that could offer new insights into seed longevity regulation and over which an optimized AA test could be beneficial to assess ecological dynamics [2]. Artificial aging tests may be useful to test seed lot status and predict when the seeds may reach the viability threshold that may require further actions to conserve the species’ germplasm [18], thus improving genebanks’ management for ex situ germplasm conservation [19]. Non-crop species’ germplasm conservation may be important for maintaining biodiversity’s option value (i.e. the value of potential future benefits that are unknown today, such as finding new pharmacologically-beneficial substances), in spite of the difficulties of defining this concept [20–22].

Invasive species constitute one of the five major drives of global biodiversity loss [23] and eradication costs increase with invasion time specially for those invaders that are able to generate a permanent soil seed bank [24]. *Carpobrotus edulis* (L.) N. E. Br. (Aizoaceae) is a mat-forming trailing succulent perennial that has been introduced into all continents, mostly growing in the Mediterranean regions [25]. This species is native from South Africa where it is used in traditional medicine for its several pharmacological applications for the treatment of tuberculosis and other respiratory infections, toothache and earache, facial eczema, wounds, burns, hypertension, and diabetes mellitus, due to the antimicrobial, antiproliferative, and antioxidant properties of *C. edulis* leaf extracts [26]. Despite its pharmacological potential, in the introduced range, this species impacts native communities by decreasing biodiversity and altering nutrient cycling dynamics [27, 28]. The production of allelopathic compounds contribute to the drastic modification of this species surroundings inhibiting native species germination [29]. One of the factors determining this invasive species’ persistence is the intense flowering and high amount of small hard-coated reniform orthodox seeds produced, which generates a permanent soil seed bank [30]. Invasive species eradication costs are extremely high in advanced invasion stages, especially because local eradication is not possible as long as seeds remain viable in the soil. In weed eradication and control programs it is critical to know the characteristics of the soil seed bank for the implementation of successful control measures and monitoring [31]. Some ex situ techniques have been proposed to predict the persistence of seeds and to enable policy makers to make faster and better-informed decisions in weed-management programs [32]. Assessing seed longevity in invasive plants becomes crucial for management policies when prioritizing zones or determining budget allocation in post-eradication plans. Considering *C. edulis* wide distribution, its ecological impacts and the fact that it is able to generate a permanent soil seed bank that increase eradication costs, we aimed to describe a rapid AA test for the seeds of this invasive species, evaluating the role of different parameters to estimate seed vigor in natural populations. We tested over different natural populations the optimal conditions that generate a progressive viability loss following the sigmoidal response described for seed aging within a short time period (less than 10 days), maximizing feasibility with low economic costs. This test may be useful for management programs to assess invasive potential, define eradication strategies and promote effective genebank management in non-crop species.

## Results

**Fig. 1:**
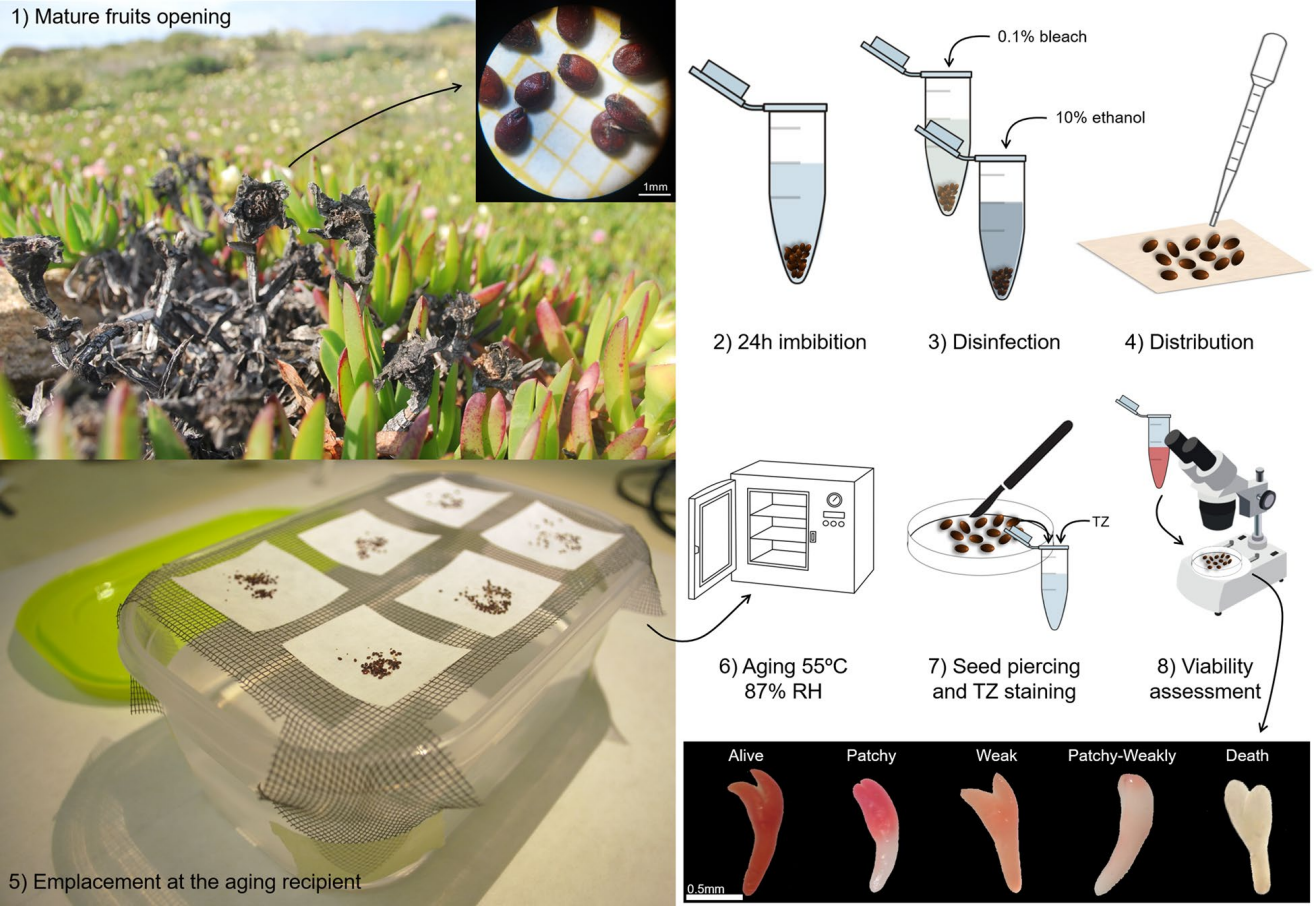
Eight-step accelerated aging test optimized for *C. edulis*. Eight steps describing the accelerated aging protocol from seed collection (step 1) to classification of stained *C. edulis* embryos after tetrazolium (TZ) incubation for viability testing according to the staining intensity and distribution (step 8)

The different experimental conditions tested lead to the understanding of the importance of several factors determining artificial aging seed survival curves in *C. edulis*. All artificial aging tested conditions lead to a loss in the initial seed viability, but its timing was strongly influenced by the induced conditions. When it comes to keeping the aging environmental stable, some treatments were found more feasible than others, which helped to decide the best artificial aging environment for *C. edulis* seeds in terms of speed, feasibility and simplicity. The final aging protocol (resumed in Fig. 1) was defined at 55 °C and 87% RH and revealed differential seed survival curves between the two tested natural populations.

### Temperature

**Fig. 2:**
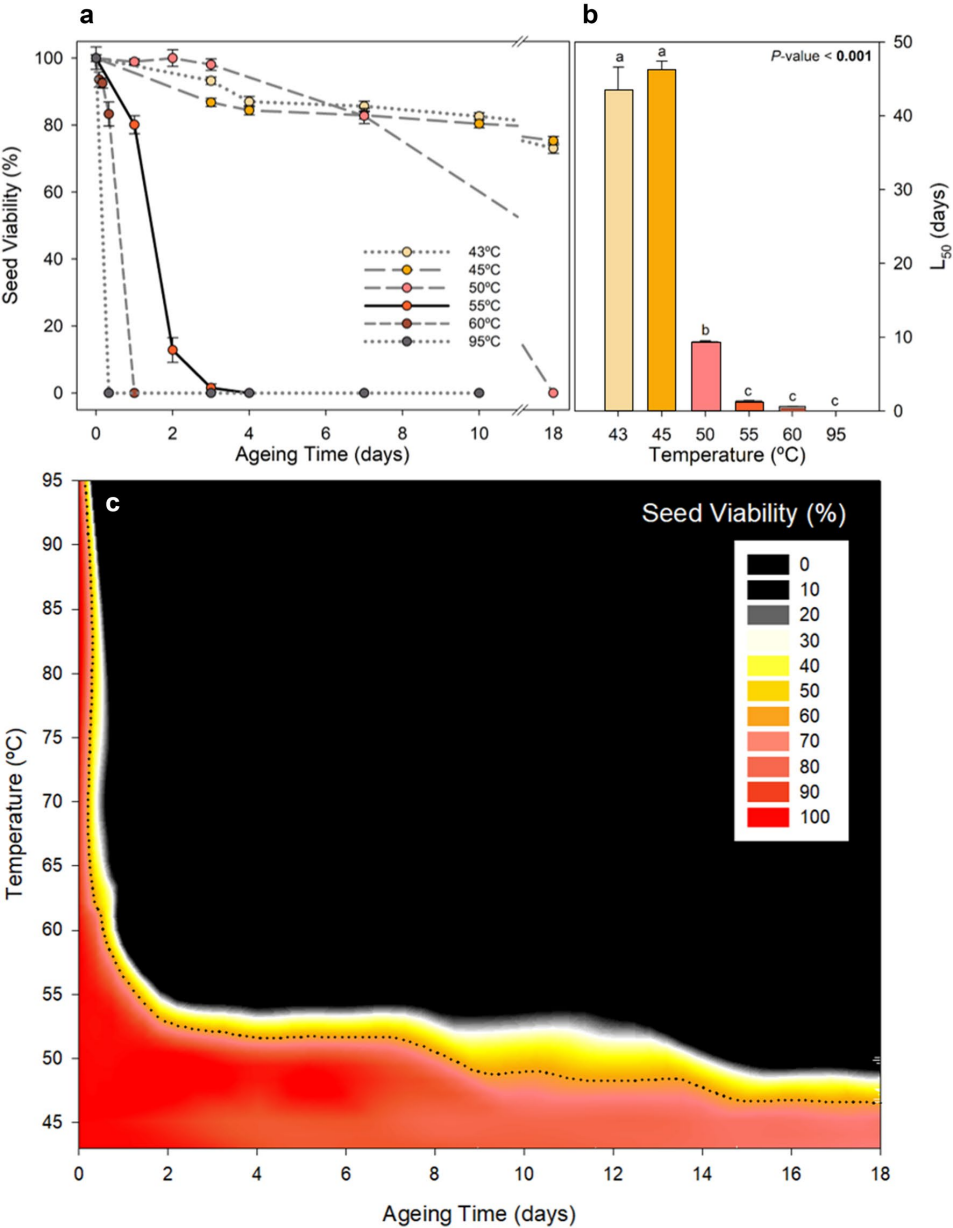
Influence of temperature and aging time in artificially aged seeds of *C. edulis.***a** Viability loss of *C. edulis* seeds at the different tested temperatures and 87% RH. Data is represented as Mean ± SE (n = 6). **b** L_50_ (loss of 50% viability) at the different tested temperatures. Different letters indicate statistically significant differences (*P*-value < 0.05). **c** Contour plot of seed viability considering temperature and aging days. Dotted line represents the L_50_, 50% viability loss

Temperature strongly influenced seed survival curves and their medium longevity (Fig. 2). The lowest tested temperature (43 °C) led to a constant and unhurried loss of seed viability of only a 20% loss in 18 days (Fig. 2a). Similar aging pace was observed at 45 and 50 °C but an abrupt change was observed at 55 °C, leading to the typical sigmoidal seed survival curve (Fig. 2a). Higher temperatures induced 100% viability loss between 3 and 24 h under 60 °C and less than 3 h under 95 °C (Fig. 2a). Those temperatures led to the apparition of burned embryos with brownish coloration. The L_50_, the time required to reach a 50% viability loss decreased sharply with increasing temperatures, from around 45 days under 43-45 °C to just a few hours at 60 °C (Fig. 2b). Temperatures above 55 °C may offer artificial aging intervals within a week, as L_50_ was found between 2 and 3 days of aging treatment. Seed viability did not decrease linearly with temperature and aging time, instead, a strong inflection point was observed around 55 °C when contrasting aging time, temperature and seed viability (Fig. 2c).

#### Relative humidity and seed imbibition

**Fig. 3:**
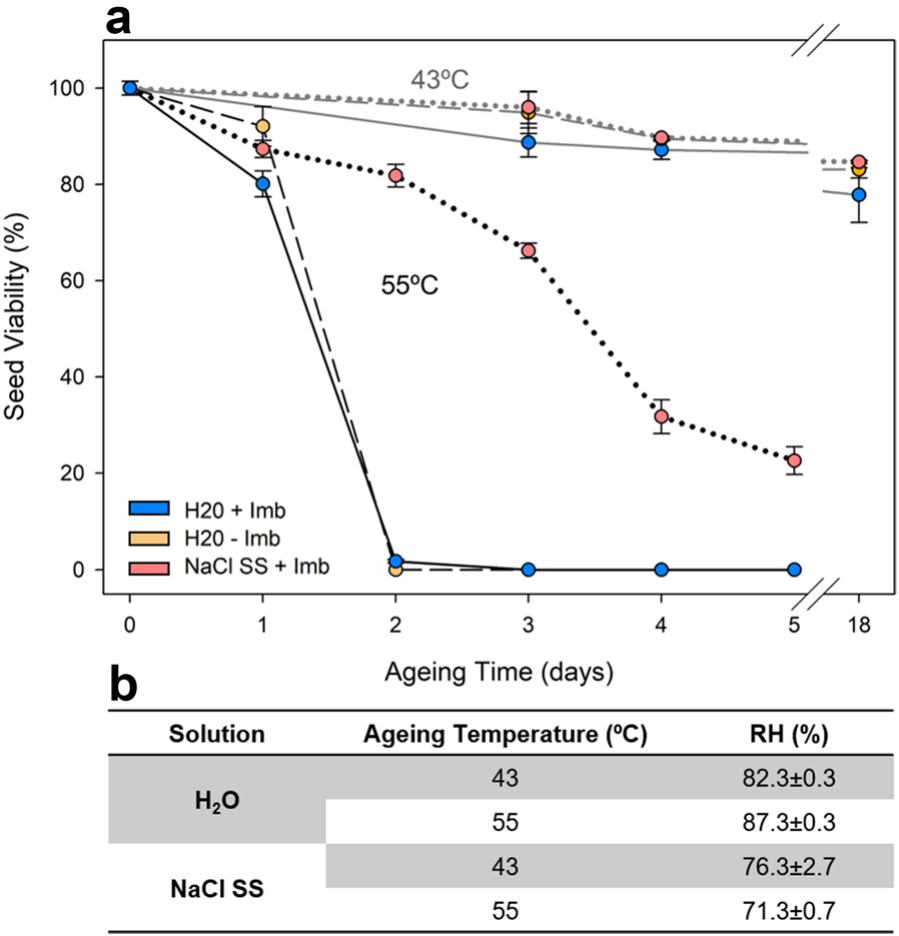
Relative humidity in interaction with temperature and imbibition effect in aging seeds of *C. edulis*. **b** Temperature and RH conditions in the aging recipient for the different tested conditions. NaCl SS: NaCl saturated solution. Data is represented as Mean ± SE (n = 6). Temperature, Aging time and Relative humidity effects were strongly significant (*P*-value < 0.01), whereas imbibition had non-significant effects (*P*-value = 0.328)

The amount of water vapor in the aging atmosphere determined differences in seed survival curves. The superior RH obtained in the 82-87% RH experiment (using distilled water) determined an accelerated viability loss in contrast with the 71–76% RH experiment (using a saturated salt solution, NaCl) (Fig. 3). This difference was observed under 55 °C but not at 43 °C, where no differences in the seed survival curve were observed (Fig. 3). When contrasting treatments at 55 °C, high RH (87%) lead to a complete viability loss after two aging days, whereas a lower humidity (71%) triggered a progressive viability loss that leave 20% viable seeds after five aging days (Fig. 3). Apart from a different seed survival curve, the imbibed seeds in low RH conditions (71%) at 55 °C were found with a hardened seed coat at all aging times, hindering seed piercing for viability analysis.

Seed imbibition did not exert any significant effect on seed survival curve at any aging time when compared with non-disinfected dry seeds (Fig. 3a). However, clear practical benefits were found when placing seeds on the aging container with water using a Pasteur pipet, instead of placing them one by one in dry with tweezers. In spite of the fact that imbibed seeds and non-disinfected dry seeds showed no significant differences within the viability loss interval (0–120 h), the lack of seed disinfection promoted fungus attacks from the 7th aging day in some replicates, which contributed to discard this treatment.

#### Viability and germination’s potential loss

**Fig. 4:**
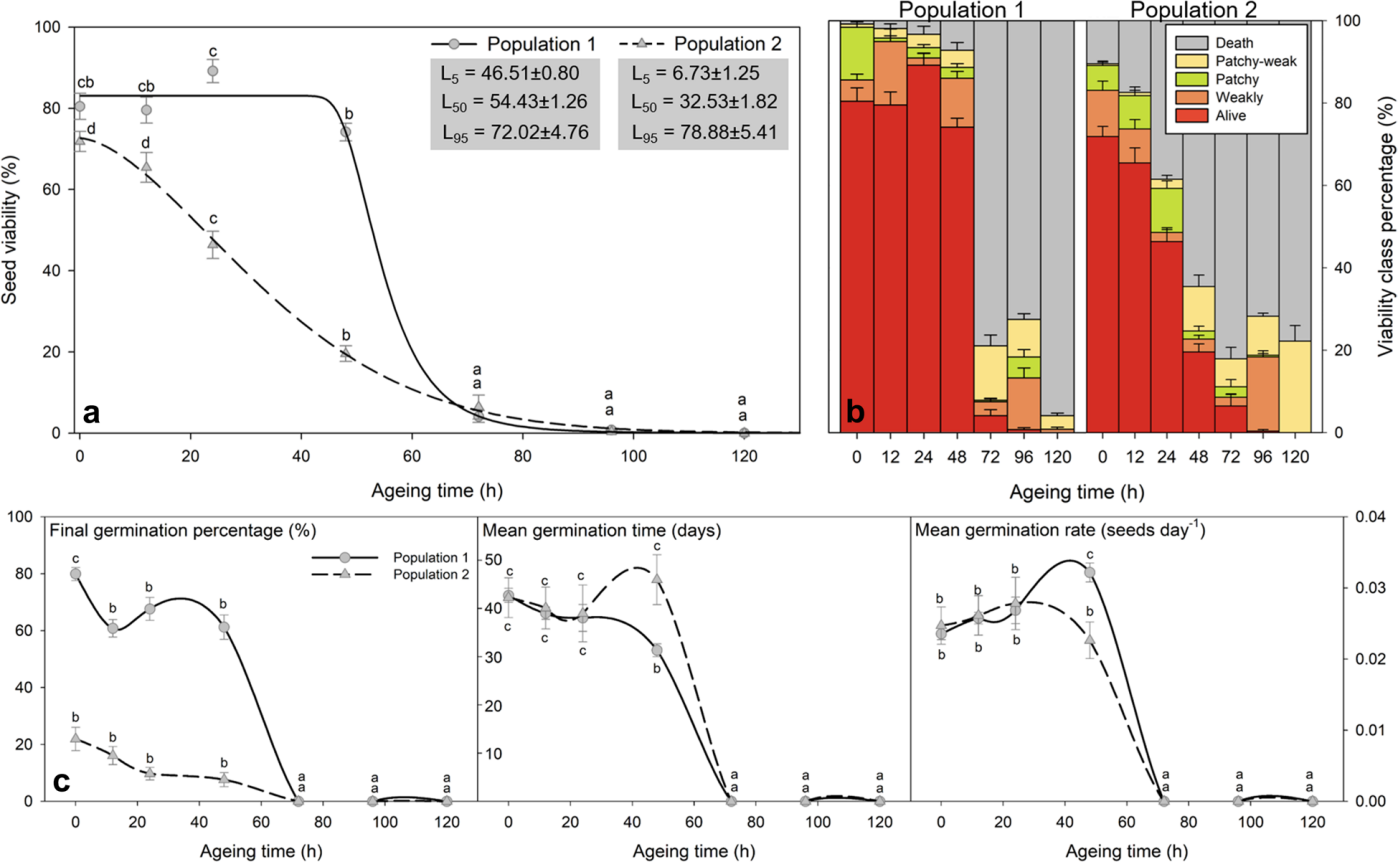
Viability and germination loss with aging time at 55 °C and 87% RH of *C. edulis* seeds from two natural populations. **a** Viability loss and obtained values for L_5_, L_50_, L_95_ for each population. Black lines correspond to best fitted Weibull model for each seed lot (n = 35). **b** Percentage of embryo classes during aging in *C. edulis* seeds considering the two populations. **c** Germination parameters variation with aging time. Different letters in **a** and **c** indicate statistically significant differences (*P*-value < 0.05) within a population. Data is represented as Mean ± SE (n = 5)

The seed survival curves in terms of viability and germination tested in the two different populations of *C. edulis* triggered two differentiated responses. Initial seed moisture content was not significantly different (*P*-value > 0.05) between the two populations: with 13.54 ± 0.82% and 14.22 ± 0.64% in population 1 and population 2 respectively. Population 1 followed a sigmoidal pattern with an abrupt fall within the 40 and 80 aging hours range, whereas population 2 showed a slow viability loss without a resistance phase (Fig. 4). Initial viability was found very high (around 70–90%), whereas initial germination percentage fell to 20% in Population 2. The viability loss was accompanied by an increase in the proportion of dying seeds in both populations (patchy and patchy-weakly embryos, Fig. 4b). Those dying seeds were unable to germinate as shown by the null germination rates found after 72 aging hours (Fig. 4c). Germination time was initially similar between the two populations and maintained at 40 days until 24 aging hours. Whereas population 1 maintained mean germination time after 48 aging hours, population 2 showed a significant decay at this time. Similarly, a strong decrease in the mean germination rate was observed past the 48 aging hours (Fig. 4).

#### Seed longevity estimation

A non-linear response was found for viability loss with aging time (Fig. 4a). The best fit for the sigmoidal curve of the seed survival was observed using Weibull model adjustment. This model allowed the estimation of different parameters (Fig. 4a). The best fitted viability loss for population 1 was a Weibull type 2 with three parameters:

$$V = 83.06 \left( {1 - e^{{ - e^{{ - 9.3\left( {\log \left( x \right) - \log \left( {52.33} \right)} \right)}} }} } \right)$$

Whereas the best-fitted viability loss for population 2 was a Weibull type 1 with three parameters:

$$V = 72.64 e^{{\left( { - e^{{1.65(\log \left( x \right) - { \log }\left( {40.61} \right)}} } \right)}}$$

where V stands for Viability (%), and x for aging time in hours. To characterize seed survival curve we propose the use of three different parameters that reflect: aging resistance (L_5_), mean longevity (L_50_) and lethal aging time (L_95_) under aging conditions, that define the number of hours to achieve 5, 50 and 95% of viability loss respectively. Those parameters characterize the two differentiated seed survival curves obtained for the two natural populations. The lack of seed resistance to aging conditions of population 2 was reflected in the small L_5_ (6.73 ± 1.25 h) in comparison with population 1 (46.51 ± 0.80 h) (Fig. 4). Moreover, different L_50_ reflected the medium seed longevity that also differed between populations and was found between 32 and 54 aging hours. Lethal aging time (L_95_) was found past the 72-79 aging hours in both populations.

## Discussion

### Seed resistance to temperature and relative humidity

Temperature played the most important role in reducing seed longevity in *C. edulis*. Increasing temperatures are described to reduce the accelerated aging time, with seeds reaching the 0% viability in a shorter time [11]. Substantial decrease in the activity of antioxidants in response to increased accumulation of reactive oxygen species is associated with accelerated aging [33–36]. If not counterbalanced, reactive oxygen species lead to the accumulation of damaged molecules such as lipid hydroperoxides and protein carbonylation that increase strongly in deteriorating seeds and may ultimately lead to seed death [37, 38]. Seeds may respond to high temperatures with seed death (non-tolerant seeds), non-altered viability (tolerant seeds) or trigger increased germination (i.e. pyrophile plants) [39, 40]. Seed survival curves may vary depending on specific temperature and exposure time [41]. In the case of *C. edulis*, 5 °C increase from 45 to 50 °C were enough to reduce 4 times the L_50_, suggesting a strong sensitivity to temperature. Moreover, *C. edulis* seeds did not tolerate 3 h exposure at 95 °C as other Aizoaceaceae may do according to the study developed by Daws et al. [42], where at least 20% seed survival was observed in 26 Aizoaceae species at 103 °C for 17 h. Seeds of *Welwitschia mirabilis* from the Namib Desert have been reported to tolerate 80 °C for 48 h [43]. However, those studies were orientated to evaluate seeds thermo-tolerance and low RH was used. High RH (up to 75–80%) is recommended for testing seed longevity [8]. Differences in RH generate differential seed survival curves in *C. edulis* seeds under sensitive temperatures, with higher RH (87%) promoting a faster seed aging in comparison with lower RH (73%). This result coincides with those obtained in *Jatropha curcas* L. seeds, which showed a faster deterioration in AA tests over water in contrast with using a saturated salt solution [44]. Similar results were obtained with *Hirschfeldia incana* when comparing humid (75%) and dry (33% RH) storage at 35 °C, where humid storage decreased almost 100 times seed longevity [45]. In fact, resultant products of non-enzymatic modifications of protein structures through glycation found in accelerated aged soybean seeds were formed most rapidly in seeds at high humidity [46]. The inactivation of proteins would depress the metabolic capacity and reduce the ability of biological systems to repair cellular damage occurring during storage [47].

When contrasting aging tolerance of *C. edulis* seeds with conventional artificial aging protocols for commercial seeds, *C. edulis* appears to have an increased tolerance. Considering that the general temperature for artificial aging is between 41 and 43 °C for 48 to 72 h [8, 15], *C. edulis* seeds may be considered resistant, as under those temperatures, viability was maintained above 80% at least for 10 days. Moreover, 80% of *C. edulis* seeds survived after a heat shock of 3 h at 60 °C, whereas imbibed seeds from other species may not even survive a 30-min shock at this temperature [48]. *Ex*-*situ* seed longevity was found to be correlated with drought exposure during the post-zygotic phase in dry and warm environments using 16 closely-related populations of the genus *Silene* [49]. Post-zygotic *C. edulis* phase occurs during summer, coinciding with low water availability and the highest yearly temperatures, [50] which may contribute to explain increased ex situ seed longevity in comparison with other commercial species subjected to similar AA tests.

### Optimal conditions for artificial aging and measurement of seed longevity

The optimal conditions for AA test in *C. edulis* were selected considering speediness, simplicity and sensitivity. The chosen conditions for artificial seed aging were 55 °C over water (87% RH) using imbibed *C. edulis* seeds. The chosen temperature and relative humidity offer the possibility to test seed longevity within a week, as longevity is lost in 3–5 days. Moreover, 55 °C constitutes the inflection point in the viability loss-aging time curve suggesting high sensitivity to changes in seed longevity at this temperature. Besides the advantageous temporal scale of using 55 °C and a water solution, it also requires less preparation than using a saturated salt solution and avoids the risk of salt crystallization over seeds. Finally, seed imbibition and disinfection did not lead to any significant difference and offered substantial practical benefits as seed softening, disinfection facilities and seed disposal in the aging containers pipetting seeds instead of nipping them one by one.

Seed survival curve typically shows a lag phase before seed viability starts to decline rapidly [8]. This was the observed response for *C. edulis* seeds to selected artificial aging conditions, in both the analysis of seed viability (which took two working days in a 3 days’ time interval) and seed germination (which took 70 days of germination control every 3 days) coinciding in duration of the lag phase and survival loss in the two *C. edulis* populations. Viability measures may be equally accurate and may be less time-consuming than germination measurements, as also exposed in Xu et al. [51]. Although both populations showed similar seed viability (around 70-80%), population 2 showed a 25% germination, revealing that 45% of viable seeds did not germinate, which may correspond to dormant seeds. Viability test is also preferable in comparison to germination test as it reflects seed lot viability regardless of their dormant state [52]. The characterization of the tipping point in the modeled sigmoidal curve of viability loss before the sharp decline is essential, corresponding to exhaustion after the resistance phase in seed stress responses, characterized by a failure of protection and repair and critical cell death [53]. To characterize the duration of the resistance phase, L_5_ (the aging time when 95% of the initial viable seeds remain viable) is here proposed. Company et al. [4] have proposed P_20_ (the aging time to decrease a 20% seed survival) to characterize the first survival loss in the curve but this measure may fall very close to the L_50_ and will not provide new information. The aging time that causes a 50% decrease in seed survival has been largely accepted as a measure of seed longevity [8]. Besides this measurement, the point where seeds no longer germinate (P_0_) was proposed by Dowsett et al. [2] to be a better measurement of seed longevity due to the rapid decline in germination. In this way, Company et al. [4] also proposed the use of P_80_ (80% of seed mortality). We suggest that L_95_ (the point where only 5% viability is observed) may be a better indication of the lethal seed aging time when estimating parameters from a fitted curve. The triplet of parameters: L_5_, L_50_ and L_95_ may provide information on sensitivity, medium aging time, and maximum resistance of a seed lot.

### Artificial aging to assess invasive potential

Dowsett et al. [2] suggested that artificial aging tests may have a valuable role in understanding the biology of weed species. Besides understanding their biology, artificial aging tests may contribute to design effective management policies and eradication strategies. Recently, Company et al. [4] evaluated the potential competitive displacement of the invasive species *Cortaderia selloana* over the native *Saccharum ravennae* using seed germination and viability tests under AA conditions and revealed that no long periods of subsequent monitoring are needed after eradication of *C. selloana*, as this species presented low seed longevity. The two *C. edulis* populations where the AA test was tested showed different seed longevities towards an increased seed vigor in population 1, that may require increased eradication efforts. Accelerated aging tests on *C. edulis* may allow rapid assessment by managers and policy makers of seed’s vigor at different zones and may contribute to design effective monitoring programs taking into account seed longevity estimations with appropriated budget allocation. Moreover, post-eradication soil seed bank vigor monitoring may contribute to define re-invasion risks and estimate weed eradication programs [32].

The artificial aging test optimized contributes to the understanding of invasive seed thermosensitivity that may be used to design soil seed bank eradication treatments. In this way, soil solarization is a non-chemical environmentally-friendly agricultural method for soil disinfestation. Using transparent polyethylene sheets, the temperature of the soil at a depth of 0–20 cm usually reaches 40–60 °C, leading to the eradication of pathogens, arthropod pests, and weeds. This soil treatment has been already tested to manage invasive seed banks and lead to significantly reduced viability of buried *Acacia* seeds, including the invasive species *A. saligna*, *A. murrayana* and *A. sclerosperma*, by exposing seeds to lethal hydrothermal conditions, constituting an effective method to reduce the persistent invasive seed bank of those species [54–56]. Considering the *C. edulis* seeds’ thermic sensitivity described here and the fact that a 77.6% of this species’ soil seed bank is mainly found in the species’ litter and the first 0-5 soil cm [30], soil solarization may constitute a promising tool for invaded soil seed bank eradication. Just 24 h at 60 °C and 87% RH may be enough to obtain a complete viability loss in *C. edulis* seeds. Midday soil surface temperatures in the range 50–80 °C have been reported in environments ranging from tropical forests to deserts [57, 58], and 60 °C may be easily obtained under polyethylene sheets with air temperature reaches 30 °C [56]. However, further research is needed to determine the effectivity of a soil solarization treatment under natural soil moisture conditions and the effect of thermal cycles versus the sustained temperatures explored here.

## Conclusions

The inflection point found with temperature-aging time analysis led to the definition of a rapid and sensitive method for *C. edulis* seeds artificial aging at 55 °C under 87% RH, where viability is lost within a working week, allowing managers to easily test seed vigor and longevity to assess invasive potential and contribute to design effective monitoring programs taking into account seed longevity estimations with appropriated budget allocation at different invaded areas. *C. edulis* seed thermosensitivity has been addressed suggesting that soil solarization treatments that mimic the described conditions may be effective for this species’ soil seed bank eradication. The optimization of artificial aging tests in non-crop species may provide new insights into seed longevity regulation and develop efficient management practices for ex situ germplasm conservation.

## Methods

### Plant material

A first lot of *C. edulis* seeds was selected to test the aging conditions from a natural population growing in Premià de Mar (Spain, 41° 29′ 24.2″ N 2° 21′ 49.3″ E). Once aging conditions were set, seeds from two natural populations of *C. edulis* were selected because of their large number of individuals and their large seed availability (population 1: 41° 28′ 25.5″ N 2° 17′ 54.7″ E; population 2: 41° 28′ 30.3″ N 2° 18′ 04.6″ E). These populations were used to test the accelerated aging test. From each population, within the period of natural seed rain, 600 fruits were collected to ensure representativeness. Fruits were opened and all the obtained seeds were pooled (more than 10.000 seeds per population). Seeds were kept at darkness and room temperature until analysis (3–4 weeks). Prior to start the accelerated aging tests, seed moisture content (%) was measured for both populations as: FW − DW*100/DW, where FW stands for 50 seeds fresh weight and DW for those 50 seeds dry weight (obtained after 72 h at 70 °C) with five replicates. In the two populations, accelerated aging through viability tests and germination was tested as described in the following sections.

### Accelerated aging test

Different containers were tested regarding their surface/volume ratios to maximize seed disposition and effectivity and preservation of the aging conditions. It was concluded that a commercial polypropylene hermetic container of 1.1L capacity with a 20 × 5 cm surface (PlasticForte, Spain) was ideal to generate a controlled atmosphere that maintains the desired RH and hold the seeds at a determined point with 70% of the capacity filled with aging solution (Fig. 1). There was an exception at 95 °C where due to the temperature, the experiment was performed using glass jars. Five replicates per population, including fifty seeds per replication were used.

The preparation of the aging containers was performed as follows: first, containers and sized meshes were disinfected with 70% ethanol and filled with 700 ml of aging solutions: water or NaCl saturated solution (See relative humidity experiment below). Mesh was fixed using masking tape. Seeds were submerged in 0.1% bleach for 30 s and 10% ethanol for 30 s more and washed multiple times with distilled water. Containers were only opened when the aging treatment ended at the chosen time: from 3 to 18 days. At that moment, seeds were prepared for the viability test as described in the succeeding sections. Zero aging hours corresponds to viability (through tetrazolium viability test) or germination assessment on non-artificially aged seeds for viability and germination tests respectively.

### Temperature experiment

The role of aging temperature, RH and seed imbibition and disinfection on the seed survival curve in terms of seed viability was tested. The general aging procedures suggest the use of 41–43 °C in a high humidity environment for a specified duration (48–72 h) [8, 15]. However, seeds from some Aizoaceae species are known to resist even higher temperatures for a short period of time [42]. Considering this, aging was tested at the following spectrum temperatures: 43, 45, 50, 55, 60, 95 °C using a laboratory oven under high humidity conditions using distilled water aging solution inside the containers that generated an atmosphere of 87% RH.

### Relative humidity and seed imbibition experiment

To evaluate the role of RH inside the aging container, distilled water and a saturated commercial NaCl solution (40 g/100 ml H_2_O) were used. Saturated salt solutions are often used for controlling environmental variables as they generate a defined RH environment, inferior to a water-saturated atmosphere [44]. Relative humidity and temperature inside the aging container were tested by introducing a data logger sensor (EasyLog, Lascar Electronics) and a wireless digital thermometer and hygrometer (WA10-Kira EU, Oria) after different time intervals (24, 48, 72 and 96 h inside aging containers).

Seed imbibition is recommended in tetrazolium viability tests (see seed viability section) because it softens the seed coat and activate enzymatic systems [52]. In order to standardize conditions for initial viability measurement and accelerated aging conditions, imbibition was also proposed before accelerated aging. Seed imbibition was performed with distilled water during 24 h (for its compatibility with the normal laboratory routine) in the dark and at room temperature. Unlike imbibed seeds, dry seeds were not disinfected to maintain the dry conditions and compare the seeds dry original state and the imbibed and disinfected seeds. Using a Pasteur pipette for imbibed and tweezers for dry seeds, seeds were distributed into the 10 cm^2^ filter paper that was placed on the containers’ mesh together with other replicates.

### Seed viability

To asses seed viability at the different aging times, the tetrazolium (triphenyl tetrazolium chloride) test was used [15, 52, 59]. Seeds were delicately pierced under a binocular magnifying glass to ensure tetrazolium penetration. No staining was observed in non-pierced seeds. Five replications per population, including fifty seeds per replication were disposed in 2 ml capacity eppendorfs with 0.5 ml of 0.1% tetrazolium (Sigma-Aldrich, Steinheim, Germany) and was incubated for 48 h at 40 °C before viability assessment (Fig. 1). Tetrazolium incubation conditions were chosen following the recommendations from standard procedures [15, 52, 59] that suggest higher temperatures (within the defined accuracy range 20–40 °C) to speed up the staining process. Tetrazolium staining time was evaluated within the interval described for some species from 2 to 48 h [52, 59] and staining time inferior to 48 h revealed incomplete stained embryos. After two days, tetrazolium was withdrawn using a 1 ml pipette and 0.5 ml of distilled water was added to each eppendorf to facilitate further seeds displacement. Using a Pasteur pipette, seeds were spread in a petri dish under a binocular magnifying glass and seed viability was assessed using tweezers. A little squeeze was enough to force the embryo to escape the seed coat and determine its vigor. Six different categories were used to classify embryo status: viable (totally stained), weakly (pale stained), patchy (partially stained), patchy-weakly (partially pale stained), death (white embryo), and aborted (no embryo found) (Fig. 1). The sum of weakly, patchy and patchy-weakly seeds constitutes low vigor seeds without survival probabilities (dying). To speed and facilitate data collection, three smart phone free apps were used for voice recording during embryo viability classification (Voice recorder from Splend Apps), afterwards fast reproduction (Music Speed Changer from Single Minded Productions, LLC), and class counting (Thing counter from Karuma).

### Seed germination

Seed germination was performed as described in Novoa et al. [60] and Podda et al. [61] with brief modifications on artificially aged seeds of populations 1 and 2 for the different aging times (0, 12, 24, 48, 72, 96 and 120 h). Different conditions of pH, temperature alternation and acid pre-treatment were tested, obtaining the best results with 12 h photoperiod with temperature alternation between 25 °C during the day and 10 °C at night. Germination was assessed every three days over wet Whatman paper (90 mm No. 1 filter) in petri dishes. Five replications per population, including fifty seeds per replication were used. Final germination percentage was estimated after curve saturation at 70 days. Mean germination time and mean germination rate were estimated using the *GerminaR* package in R 3.5.0.

### Data analysis

Significant differences between aging time or temperature were tested using a one-way ANOVA, evaluating the effects of the fixed factor ‘Aging time’ for the different variables in Fig. 4 and the fixed factor ‘Temperature’ in Fig. 2b using Tukey test to contrast differences between factor levels in R 3.5.0. A four-way ANOVA was used to test the effects of the three fixed factors: ‘Relative humidity’, ‘Temperature’, ‘Aging time’ and ‘Imbibition’, for the times 0, 24, 48 72, 96 h, assuming that viability at 43 °C after 24 and 48 h was equal to 72 h in Fig. 3a. Normality and homoscedasticity were confirmed using the Shapiro–Wilk and Levene tests respectively. Seed survival curves were fitted using the *drc* R package for dose–response curves fitting that include different adjustments, including Weibull models that can contemplate asymmetrical losses’ responses [62]. The best model was selected using the AIC criteria. Longevity parameters (L_5_, L_50_ and L_95_) were estimated using the ED function from the *drc* R package. SigmaPlot 10.0 (Systat, USA) was used to create the presented plots.

## Data Availability

The datasets used and/or analyzed during the current study are available from the corresponding author on reasonable request.
